# Selenium-Enriched Brushite: A Novel Biomaterial for Potential Use in Bone Tissue Engineering

**DOI:** 10.3390/ijms19124042

**Published:** 2018-12-14

**Authors:** Aleksandra Laskus, Anna Zgadzaj, Joanna Kolmas

**Affiliations:** 1 Department of Analytical Chemistry and Biomaterials, Analytical Group, Medical University of Warsaw, ul. Banacha 1, 02-097 Warsaw, Poland; alaskus@wum.edu.pl; 2 Department of Environmental Health Sciences, Medical University of Warsaw, ul. Banacha 1, 02-097 Warsaw, Poland; anna.zgadzaj@wum.edu.pl

**Keywords:** selenium, brushite, dicalcium phosphate dihydrate, ionic substitution, biomaterials, bone substitutes

## Abstract

In this study, a novel biomaterial, i.e., brushite containing 0.67 wt% of selenium (Se-Bru) was synthesized via a wet precipitation method. Pure, unsubstituted brushite (Bru) was synthesized via the same method and used as a reference material. Different techniques of instrumental analysis were applied to investigate and compare physicochemical properties of both materials. Fourier-Transform Infrared Spectroscopy confirmed the chemical identity of both materials. Scanning Electron Microscopy (SEM) was used to study the morphology and indicated that both samples (Bru and Se-Bru) consisted of plate-like microcrystals. Powder X-ray Diffraction (PXRD) showed that Bru, as well as Se-Bru were crystallographically homogenous. What is more, the data obtained from PXRD studies revealed that the substitution of selenite ions into the crystal structure of the material had clearly affected its lattice parameters. The incorporation of selenium was also confirmed by solid-state ^1^H→^31^P CP MAS kinetics experiments. Additionally, studies on the release kinetics of the elements forming Se-Bru and preliminary cytotoxicity tests were conducted. This preliminary research will favor a better understanding of ionic substitution in calcium phosphates and may be a starting point for the development of selenium-doped brushite cements for potential use in bone tissue impairments treatment.

## 1. Introduction

Calcium phosphates (CaP) are widely applied biomaterials in bone tissue and dental surgery. Due to their good bioactivity and biocompatibility, they serve as bone fillers, coating materials and drug delivery system matrices [1]. One of them, dicalcium phosphate dihydrate (DCPD), described with the formula CaHPO_4_·2H_2_O, and known as a mineral brushite, exhibits relatively high solubility [2]. Hydrolyzing easily to octacalcium phosphate and hydroxyapatite, DCPD is considered to be an intermediate phase in bone mineralization and enamel dissolution [3]. Therefore, it is frequently used as a moldable, ready-to-use bone cement and dental coating [3,4].

The solubility of synthesized biomaterial affects the regeneration of mineralized tissue and the release rate of therapeutic agents, i.e., foreign ions or drugs, which may be introduced into the crystal lattice of CaP. Introducing foreign ions involves both cationic and anionic sites of the crystal lattice and is one of the ways of improving different properties of the biomaterials. Not to be groundless, silicon and magnesium are frequently used to enhance the bioactivity of the material [3], zinc and silver improves effectively its antibacterial activity [5], while iron and selenium play a key role as antitumor agents [6,7].

Being an important constituent of about 25 selenoproteins, selenium takes part in oxidative stress protection and positively affects the immune system and cell proliferation. It is also essential for bone health: a lowered selenium intake may lead to reduced bone turnover and bone mineral density (BMD) [6,8]. What is more, a large number of studies has indicated that selenium exhibits a significant anticancer activity. In this field, the form of selenite (SeO_3_^2−^) was proved to be the most active among all other selenium species [9]. Hydroxyapatite (HA) enriched with selenite ions was found to be a promising material in the treatment of bone tissue metastases and tumors [9,10,11,12,13,14,15]. Some studies have also confirmed the antibacterial activity of selenium substituted HA [14,16,17]. Thus, introducing selenium ions into CaP crystal lattice seems very promising in a view of the development of innovative biomaterials for the treatment of different bone tissue impairments.

To our knowledge, there have been no reports in the literature on DCPD containing selenium. In this work, Se-substituted brushite was synthesized via a standard, wet method. Due to its well-confirmed anticancer potential, the selenite form was chosen as an ionic modifier. Afterwards, the biomaterial was characterized by using the following methods: Powder X-ray Diffractometry (PXRD), Scanning Electron Microscopy (SEM), Infrared Spectroscopy (FT-IR), Inductively Coupled Plasma Mass Spectrometry (ICP-MS) and solid-state Nuclear Magnetic Resonance Spectroscopy (ssNMR). Additionally, the release kinetics of the elements forming Se-Bru and the cytotoxicity of the material were evaluated.

## 2. Results and Discussion

**Figure 1:**
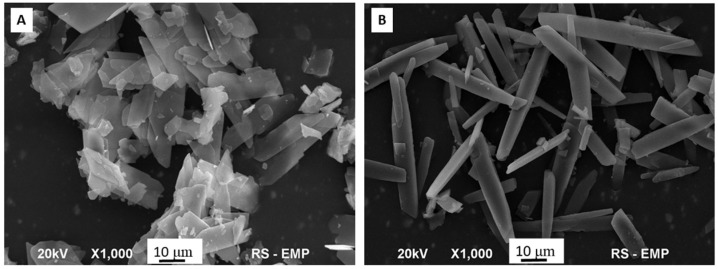
SEM images of Bru (**A**) and Se-Bru (**B**).

The SEM microphotographs (see Figure 1A,B) revealed that the obtained samples exhibited significantly different morphology. Elongated and plate-like, Bru crystals (Figure 1A) possessed a diameter and length of around 10 and 20–30 μm, respectively, whereas rod-like Se-Bru crystals (Figure 1B) characterized with a diameter of 5–7 μm and length of more than 25 μm. Furthermore, in contrast to the Se-Bru sample, the Bru crystals performed stronger tendency to agglomerate. 

**Figure 2:**
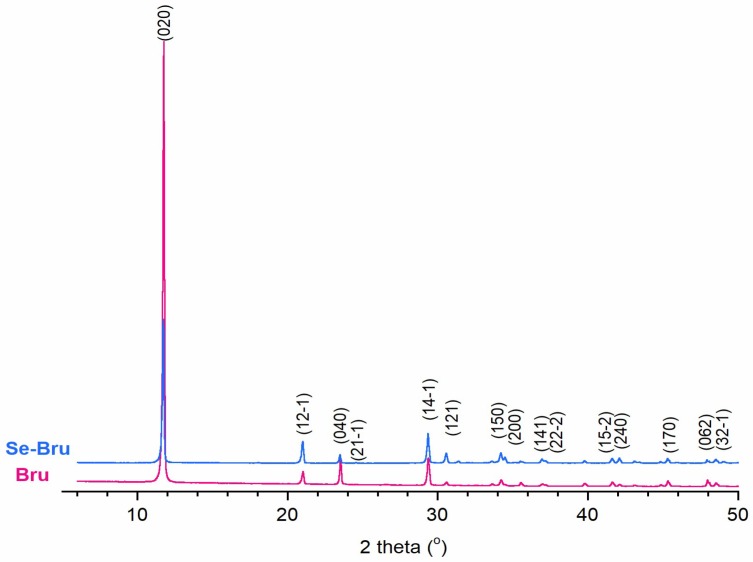
Powder X-ray Diffractometry (PXRD) patterns of Bru and Se-Bru.

The diffractograms of both samples are presented in Figure 2. All of the reflections were assigned to the brushite monoclinic structure (JCPDS 09-0077). No other crystalline phase was detected. In case of the Se-Bru sample, a slight reduction of the relative intensity of (020) and (040) reflections was observed. In comparison to pure Bru sample, these reflections varied slightly in position (see Table A1 in Appendix A), which proves that the selenite substitution into the crystal structure of DCPD affected clearly Se-Bru crystallinity and crystal morphology [15]. Additionally, the lattice parameters were calculated (see Table 1). Particularly significant increase of the *a* parameter, accompanied by the simultaneous decrease of the ***c*** parameter in case of the Se-Bru sample, confirms the incorporation of the selenite ions into the crystal structure [18]. 

The selenium content in the Se-Bru sample was measured by using the ICP-MS method (see Table 1). The concentration of Se amounted to 0.67 wt%.

**Figure 3:**
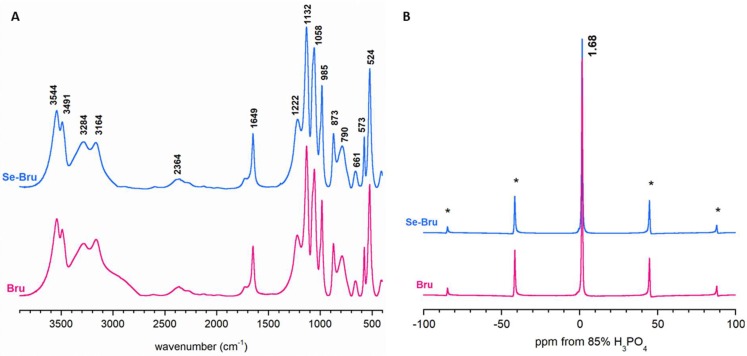
FT-IR (**A**) and 31P CP MAS NMR (**B**) spectra of Bru and Se-Bru. The main CP lines are flanked with rotational sidebands (marked with stars).

The FT-IR spectra (see Figure 3A and Table A2 in Appendix B) demonstrated the characteristic bands of dicalcium phosphate dihydrate, as it has been extensively discussed elsewhere [19,20]. Briefly, the bands in the regions of 3544–3164 cm^−1^ and 1649 cm^−1^ corresponded to the lattice water, stretching and bending vibrations, respectively. The intensive bands in the 1222–790 cm^−1^ region originated from acidic phosphate groups of DCPD, whereas the band at 661 cm^−1^ was assigned to the water libration mode. Unfortunately, the selenite bands (at ca. 700–760 cm^−1^) were undetectable [18]. It may be supposed that the selenium content (0.67 wt%) was too small to be observed as a visible band in the region of the phosphate bands (a broad intensive band at ca. 790 cm^−1^, corresponding to the –POH vibrations).

The CP MAS NMR spectra of the Bru and Se-Bru samples included one intensive and very narrow signal at about 1.68 ppm flanked by rotational sidebands (see Figure 3B). According to the available literature [21], this can be assigned to ^31^P nuclei from HPO_4_^2−^ groups. 

**Figure 4:**
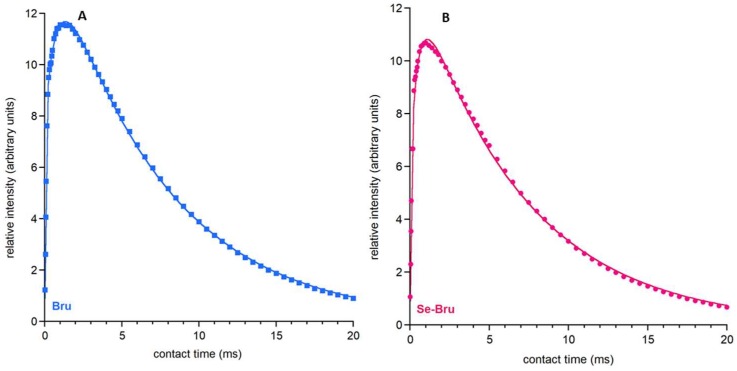
1H→31P CP MAS NMR kinetics of the studied materials: Bru (**A**) and Se-Bru (**B**).

For the signal at ca. 1.68 ppm, it was possible to analyze ^1^H→^31^P CP MAS kinetics (ν_MAS_ = 7 kHz). To achieve this goal, we studied the dependence of the relative signal intensity (peak area) I(*t*) on the contact time *t* (see Figure 4A,B). The obtained results for both samples, Bru and Se-Bru, followed a non-classical kinetic model corresponding to polarization transfer within clusters of proximate ^1^H and ^31^P spins. The experimental points were fitted to a physical function, as follows:

(1) $$I(t)=I_{0}\exp(-t/T_{1\rho }^{H})[1-\lambda \exp(-t/T_{\mathrm{df}})-(1-\lambda )\exp(-1.5t/T_{\mathrm{df}})\exp(-0.5t^{2}/T_{{\mathrm{CP}}^{*2}})]$$

where: *I*_0_ is a signal amplitude; *T*_1*ρ*_*^H^* is a proton spin-lattice relaxation time in the rotating frame; *T_df_* is a time constant of proton spin-diffusion; T_CP*_ is a CP time constant in the non-classical CP model (1/T_CP*_ is the CP rate), which characterizes the polarization transfer; *λ* is a parameter specific to a cluster of ^1^H and ^31^P nuclei involved in initial CP; for a rigid lattice *λ* = (*n* + 1)^−1^, where n is the number of protons close to the observed ^31^P nucleus; however, *λ* is dependent on molecular motion, and as such should be treated as an adjustable parameter.

Our results of λ strongly agree with previous research on the ^1^H→^31^PCP kinetics of brushite [22]. We can assume that both samples are structurally similar. However, they differ in terms of their *T_df_* and T_CP*_ parameters. *T_df_* is a parameter characterizing ^1^H-^1^H dipolar interactions: the higher its value, the slower the spin diffusion goes between the protons. The T_CP__*_ parameter characterizes the dipolar P-H interactions and the polarization transfer: the higher its value, the slower the cross-polarization. Studies of the obtained parameters (see Table 2) have shown that in the Bru sample, the proton spin-diffusion process and cross-polarization are slower than in the Se-Bru sample. This has provided another argument for the changes in the core of the crystal during the substitution of selenite ions. 

The parameters determined in this experiment (especially *T_df_* and *T_CP_*) showed that the selenite ions partially substituted the acidic phosphates in the brushite crystal structure.

**Figure 5:**
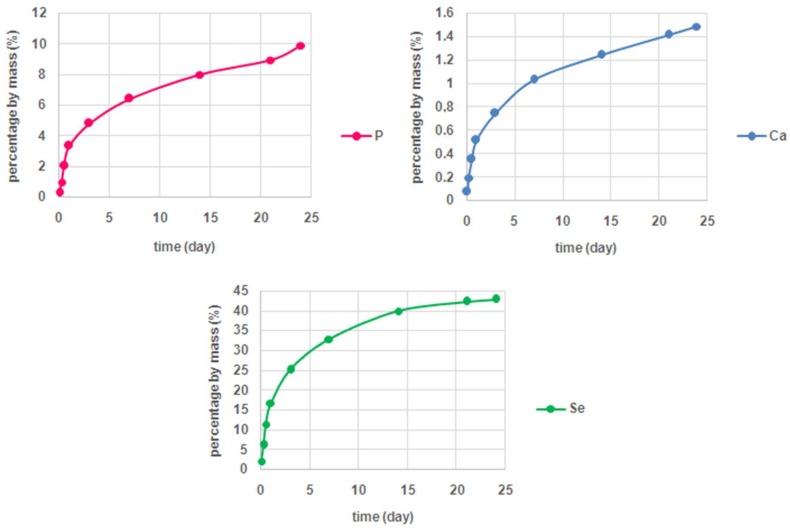
The release kinetics of the elements eluted from Se-Bru.

The investigations of the release kinetics of the ions forming the structure of Se-Bru revealed that the release rate of selenium was significantly higher than the release rate of both calcium and phosphate (see Figure 5). In case of selenium, approximately 43% of the element introduced into the structure of the material was eluted after four weeks of incubation, while for Ca and P the level of 10% of the introduced element was not exceeded. Such a suggestive difference may indicate that the selenite ions might be extensively adsorbed on the surface of brushite crystals. In turn, the low release rate of Ca and P may be explained with the slow dissolution rate of investigated CaP in the applied conditions. Taking into account a well-studied toxicity of selenium particles, such effective elution of selenite ions might be of extreme importance.

A probable reason why selenite ions can be partially adsorbed on to the surface of DCPD is the difference in spacial conformation of both SeO_3_^2−^ and PO_4_^3−^. Although both ions are characterized by a similar ionic radius (239 pm for selenite and 238 pm for phosphate), PO_4_^3−^ ions are tetrahedral, while SeO_3_^2−^ ions are of a flat trigonal pyramid conformation [22].

**Figure 6:**
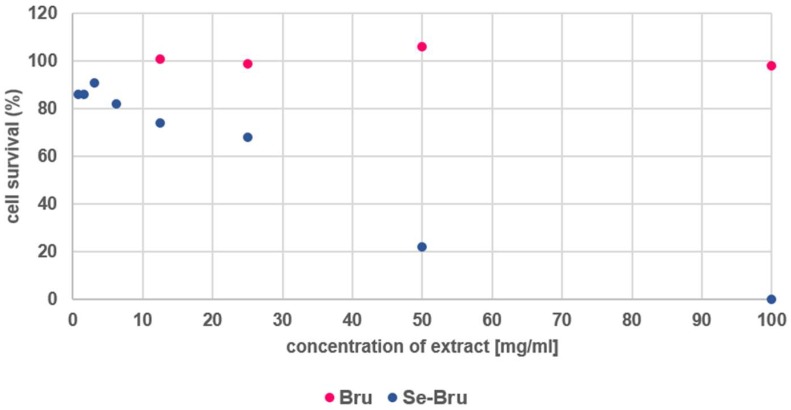
Survival of the cells after the exposition.

The results of the cytotoxicity tests correspond clearly with the release kinetics of selenium. Due to the high level of selenium eluted from the material, Se-Bru turned out to be more toxic (IC_50_ = 27 mg/mL) than the pure brushite (see Figure 6). The extract prepared with Bru was nontoxic for the cells within the whole range of dilutions, whereas the toxicity of the extract prepared with Se-Bru decreased substantially with the increase of dilution fold. This tendency may be a starting point for creating nontoxic, therapeutic CaP, modified with selenium particles. On the other hand, such activity may be of great importance in treating bone tumors. Se-DCPD could also be applied as a component of matrices for anticancer drugs targeting bones or as a filler for bone defects caused by bone tumor removal.

Studies on doping Se into the crystal structure of CaP began in 2014 and are still being conduted. Due to the triple activity of selenium particles (osteoconductive, anticancer and antibacterial) [11,12,13,14,17,23,24,25,26,27], introducing Se into CaP materials seems to be a very promising direction for bone tissue engineering. Although there have been reports on doping selenium into the crystal structure of HA [11,12,13,14,17,18,23,24,25,26,27], there has been no similar approach to synthesize selenium-modified DCPD. The possibility to introduce Se into its crystal structure has never been verified, although there are studies on doping various ions into the crystal lattice of DCPD [28,29,30]. In the studies mentioned above, Se-HA exhibited content-dependent, osteoconductive, and anticancer activity [11,13,14]. In some of them [14,17], selenium also acted as an antibacterial agent. The anticancer activity of selenium was proved to be based on the mechanism of apoptosis of cancer cells [11,13].

Hydroxyapatite is known to be the least soluble material among CaP [1]. In turn, DCPD belongs to a group of CaP, which is far more soluble than HA [1]. Due to its low solubility, bone-grafting using HA sometimes involves leaving the graft remnant in the affected area; it could lead to limited bioavailability of Ca and P for bone cells.

DCPD is most commonly used as a moldable, hydraulic bone cement with a higher surgical handiness in comparison to HA [1,31]. In addition, its solubility may improve the bioavailability of the Ca, P and other introduced, therapeutic ions, which gives it a significant advantage over HA-based, traditional biomaterials.

To briefly sum up, in comparison to the existing landscape of biomaterials, selenium-doped DCPD offers a few advantages. First, it confirms the possibility to introduce a promising agent (Se) into the crystal structure of DCPD. Second, Se-DCPD with a well-confirmed toxicity could be applied as a component of handy bone cements or matrices for anticancer drugs targeting bones, playing therefore, a synergistic role in treating bone cancer. Third, cytotoxicity tests provide important information for future investigations in this field; for example, focusing on its osteoconductive properties, the amount of introduced selenium should be below 0,67 wt% in order to obtain non-toxic material.

## 3. Materials and Methods

### 3.1. Sample Preparation

Samples were prepared by using a standard, wet precipitation method. The sources of calcium (Ca(NO_3_)_2_·4H_2_O), phosphorus ((NH_4_)_2_HPO4), and selenium (Na_2_SeO_3_) were all purchased from Sigma-Aldrich, Poznań, Poland. To synthesize brushite containing selenium (Se-Bru) the reagents were weighed out so that the molar ratios of Ca/P+Se and P/Se were close to 1.0 and 1.5, respectively. Afterwards, all of them were dissolved in distilled water. The water solution of both phosphate and selenite were added dropwise to the solution of calcium. The precipitation process was carried under continuous stirring. Once the pH was adjusted to about 6, the intensive stirring was continued for one hour. The precipitate was left for 24 h to age, then filtered and washed out several times with distilled water. Subsequently, the precipitate was dried at the temperature of 90 °C for 24 h. The route of the synthesis of pure brushite (Bru) was hardly any different: the reagents lacked in selenium source and were weighed out so that the Ca/P ratio was about 1.0.

### 3.2. Characterization

The dried powders were homogenized in mortar and characterized by using the following methods:

To determine the morphology of the crystals, SEM microscopy (JSM-6390LV JEOL microscope, JEOL LTD, Tokyo, Japan) was applied. Prior to the measurements, the samples had been covered with an Au layer.

The phase composition was analyzed by using the PXRD method (Bruker DX8 Discover diffractometer with CuKα radiation, Bruker, Madison, WI, US). The lattice parameters: *a*, *b*, *c*, and *β* angle of the unit cell, as well as the cell unit volumes were obtained from the TOPAS program (Bruker).

The presence of functional groups was determined by FT-IR spectra (Perkin Elmer Spectrum 1000 spectrometer Waltham, MA, US) recorded by using the transmission technique of the KBr tablet with a spectral range of 4000 to 400 cm^−1^.

Selenium content was measured by using ICP-MS spectrometry (Optima 3100XL, Perkin Elmer). Samples had previously been dissolved in HNO_3_ and diluted properly with deionized water. 

High-resolution ssNMR spectra (Bruker Avance 400 WB spectrometer, Bruker) were collected at 298 K, operating at 9.4 T. The ^31^P cross-polarization (CP) experiments were performed in a 4-mm probe under magic angle spinning (MAS) at 7 kHz. For single CP experiments, 2.65 μs of π/2 pulse, a 10 s recycle delay, 2 ms of contact time and 32 scans were applied. The variable contact time ^1^H→^31^P CP MAS NMR experiments were performed for 64 arrayed contact time values from 25 μs to 20 ms. The relative signal intensities were calculated using the NUTS NMR program, according to a line-fitting process. The CP kinetic functions were fitted with the KaleidaGraph 3.5, Synergy Software, Synergy, Reading, PA, USA). 

The release rate of all three elements (Ca, P and Se) eluted from Se-Bru after different periods of time was determined in the phosphate buffer solution (pH = 7,4) at 37 °C. Approximately 1 g of Se-Bru powder was suspended in 12 mL of the buffer and then subjected to shaking. Subsequently, samples of 8 mL volume were collected and further investigated. The concentration of each element was measured after 1, 6, 12, 24, 72 h, and after 1, 2, 3, and 4 weeks. All measurements were performed by inductively coupled plasma optical emission spectroscopy (ICP-OES), (iCAP 7000 spectrometer, Thermo Scientific Waltham, MA, US) and done in triplicate.

In order to evaluate cytotoxicity of Bru and Se-Bru samples, the neutral red uptake test was performed on the basis of ISO 10993 guideline Annex A1 with BALB/c 3T3 clone A31 mammalian cell line. The quantitative estimation of viable cells in tested culture was based on their neutral red uptake in comparison to the results obtained for untreated cells. Dead cells have no ability to accumulate the dye in their lysosomes. The BALB/c 3T3 cells were seeded in 96-well microplates (10^4^ cells/100 µL) in DMEM (Lonza, Basel, Switzerland) culture medium and incubated for 24 h. At the end of the incubation, each well was examined under a microscope to ensure that cells form a half-confluent monolayer. Subsequently, culture medium was replaced by the tested extracts. Extracts were prepared by incubation of tested materials (Bru and Se-Bru) in the cell culture medium (100 mg/mL) with reduced serum concentration (5%) at 37 °C for 24 h. The cells were treated with at least four dilutions of each extract in a twofold dilution series for 24 h. Subsequently treatment medium was removed. The cells were washed with PBS and treated with the neutral red medium for 2 h. Afterwards, the medium was discarded, and the cells were washed with PBS and treated with desorbing fixative (ethanol and acetic acid water solution). The amount of neutral red accumulated by cells were evaluated colorimetrically at 540 nm. Polyethylene foil and latex were used as negative and positive controls, respectively. The percentage of viable cells in each well was calculated by comparing its OD_540_ result with the mean result obtained for untreated cells (incubated in the same conditions with fresh culture medium). 

## 4. Conclusions

In this study, brushite crystals were modified with selenite ions. The synthesized DCPD contained 0.67 wt% of selenium and simultaneously exhibited a rod-like microcrystal morphology. The physicochemical methods confirmed the introduction of Se into the DCPD crystal structure. Despite this fact, the evaluation of the release kinetics of the elements forming Se-Bru revealed that the selenium particles might be also extensively concentrated on the surface of DCPD, which in turn may be responsible for its high toxicity. The cytotoxicity tests might be a starting point for creating nontoxic, but therapeutic CaP modified with selenium particles. Nonetheless, the toxicity of the synthesized material could be of significant importance in view of its killing effect towards osteosarcoma cells.

## Figures and Tables

**Table 1:** Various parameters of the obtained materials.

Parameters	Bru	Se-Bru
**Phase Composition**	100% DCPD	100% DCPD
**Unit Cell Parameters**		
***a* (*Å*)**	5.915	6.238
***b* (*Å*)**	15.12	15.16
***c* (*Å*)**	6.242	5.806
***β* (*˚*)**	116.4	116.4
**Volume ((*Å*)^3^)**	500.2	491.7
**Se Content (wt%)**	--------	0.67 ± 0.03%

**Table 2:** 1H→31P CP MAS NMR kinetics parameters.

Parameters	Bru	Se-Bru
***T*_1*ρ*_^*H*^**	7.09 ± 0.05	6.84 ± 0.08
***λ***	0.51 ± 0.01	0.54 ± 0.02
***T_df_***	0.88 ± 0.03	0.56 ± 0.04
T_CP*_	0.0809 ± 0.001	0.101 ± 0.005

## Appendix A

**Table A1:** Peak positions in PXRD diffractograms for the Bru and Se-Bru samples.

	Peak Position-2 Theta (°)	
**Peak Index**	**Bru**	**Se-Bru**
**020**	**11.75**	**11.70**
**12-1**	21.00	20.99
**040**	23.50	23.47
**21-1**	24.56	24.53
**14-1**	29.35	29.33
**121**	30.56	30.54
**150**	34.19	34.18
**200**	34.45	34.45
**141**	**37.06**	**36.90**
**22-2**	37.17	37.14
**15-2**	41.61	41.60
**240**	42.09	42.08
**170**	45.30	45.27
**062**	47.95	47.92
**32-1**	48.61	48.60

## Appendix B

**Table A2:** Infrared bands (cm^−1^) for the Bru and Se-Bru samples and their assignments [14].

Wavenumbers (CM−1)		Vibration Modes
**Bru**	**Se-Bru**	
3544-3491	3544-3491	ν_3_ H_2_O (lattice water molecules)
3283-3163	3285-3167	ν_1_ H_2_O (lattice water molecules)
2943	2945	PO-H stretching
2364	2359	
1725	1727	Combination (bending) and rotation of residual free water
1649	1650	H-O-H bending of lattice water molecules
1222	1219	δ (PO-H)
1133	1134	ν_d_(P-OH)
1058	1060	
985	985	ν_s_(P-OH)
874	873	ν(P-O(H))
790	790	δ(P-O(H))
660	660	water libration
576	577	δ(O-P-O(H))
524	523	δ(O-P-O(H))
410	412	δ(O-P-O(H))
